# The SoftHand Pro platform: a flexible prosthesis with a user-centered approach

**DOI:** 10.1186/s12984-023-01130-x

**Published:** 2023-02-08

**Authors:** Patricia Capsi-Morales, Cristina Piazza, Giorgio Grioli, Antonio Bicchi, Manuel G. Catalano

**Affiliations:** 1 SoftRobotics Lab for Human Cooperation and Rehabilitation, Istituto Italiano di Tecnologia, Genoa, Italy; 2 Research Center E.Piaggio, University of Pisa, Pisa, Italy; 3 Department of Computer Engineering, School of Computation, Information and Technology, Technical University of Munich (TUM), Garching, Germany

**Keywords:** Upper limb prosthesis, Soft robotics, User-centred approach

## Abstract

**Background:**

Among commercially-available upper-limb prostheses, the two most often used solutions are simple hook-style grippers and poly-articulated hands, which present a higher number of articulations and show a closer resemblance to biological limbs. In their majority, the former type of prostheses is body-powered, while the second type is controlled by myoelectric signals. Body-powered grippers are easy to control and allow a simple form of force feedback, frequently appreciated by users. However, they present limited versatility. Poly-articulated hands afford a wide range of grasp and manipulation types, but require enough residual muscle activation for dexterous control. Several factors, e.g. level of limb loss, personal preferences, cost, current occupation, and hobbies can influence the preference for one option over the other, and is always a result of the trade-off between system performance and users’ needs.

**Methods:**

The SoftHand Pro (SHP) is an artificial hand platform that has 19 independent joints (degrees-of-freedom), but is controlled by a single input. The design of this prosthesis is inspired by the concept of postural synergies in motor control and implemented with soft-robotic technologies. Their combination provides increased robustness, safe interaction and the execution of diverse grasps. The potential of the SHP is fully unleashed when users learn how to exploit its features and create an intimate relationship between the technical aspects of the prosthesis design and its control by the user.

**Results:**

The great versatility of the SoftHand Pro (a reasearch protpotype) permitted its adaptation to the user requirements. This was experienced by the SoftHand Pro Team during the preparation for different CYBATHLON events (from 2016 to 2020). The mixed power and dexterous hand operations required by each task of the race is inspired by everyday tasks. Our prosthesis was driven by different pilots, with different habits and backgrounds. Consequently, the hand control modality was customized according to the user’s preferences. Furthermore, the CYBATHLON tasks had some variations in this period, promoting the continuous development of our technology with a user-centered approach. In this paper, we describe the participation and preparation of the SoftHand Pro Team from 2016 to 2020 with three pilots and two different activation modalities, hybrid body-controlled and myoelectric control.

**Conclusions:**

We introduced our pilots, the implementation of the two control modalities, and describe the successful participation in all CYBATHLON events. This work proves the versatility of the system towards the user’s preferences and the changes in the race requirements. Finally, we discussed how the CYBATHLON experience and the training in the real-world scenario have driven the evolution of our system and gave us remarkable insights for future perspectives.

## Background

Losing an upper limb is a substantial impairment that leads to a reduced quality of life. An upper-limb aid represents a valid support to restore some of the lost capabilities and recover autonomy in activities of daily living, work, and social interaction [1]. Nonetheless, despite the technological development of the last two decades, amputee subjects often abandon their myoelectric prostheses or choose more simple solutions [2].

Modern bionic hands are equipped with several actuators and offer the possibility of switching between multiple grip patterns [3]. They are an attractive alternative to traditional prostheses due to their high level of anthropomorphism and dexterity [4, 5]. However, myoelectric bionic hands often require the use of external touchpads or specific muscle commands to switch between different grip patterns, which is unnatural and increases the required cognitive load. Prosthesis users prefer these solutions for light duties and social activities.

Body-powered hook prostheses are still favored for more demanding activities such as high-intensity work or filthy environments. Despite body-powered prostheses showing very limited innovations and improvements in the last century, users prefer this solution not only for its low weight and cost but also for its high reliability and control simplicity. The latter aspects were highlighted during the CYBATHLON Powered Arm Prosthetic Race [6], where body-powered prostheses outperformed more sophisticated solutions.

Several factors influence the user preference for a device over another, such as the level of amputation, cost, cultural views, work requirements, or hobbies. Sometimes, users have more than one prosthesis and choose the most suitable for a task, context, or comfort [7]. For this reason, the active involvement of the user in the design process is an essential requirement to reach a sufficient level of user satisfaction and acceptance.

A user-centered design not only includes changes in the hardware but also in the control methods to adapt the prosthesis to the users’ muscle or body conditions. The former includes modifications driven by users’ preferences and developers’ experiences while testing the prosthesis in the loop. In this paper, we present the development and evolution of the SoftHand Pro platform within the preparation and participation in the CYBATHLON Powered Arm Prosthesis Race. We have participated in both competition editions of CYBATHLON (2016 and 2020) with different pilots and different control modalities, as a result of a user-centered approach. Our technological framework was driven by three pilots, who had different personal experiences and requirements, so we adapted the hand and user interface accordingly.

**Fig. 1:**
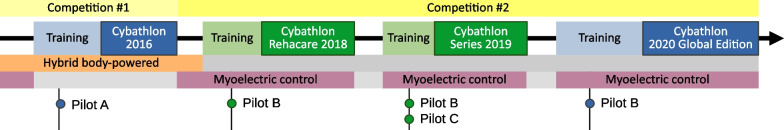
Timeline of CYBATHLON events. Chronological order of the different events in which the SoftHand Pro team has participated. The different pilots that have been part of the process of our team in CYBATHLON and the two control methods that could be selected before the training weeks before the events. Only the events in 2016 and 2020 are the official competitions. Both pilot B and C did the selection of the control method between the end of *Cybathlon 2016* and the training for *Cybathlon Rehacare 2018*

While developing a research prototype gives high flexibility, the preparation and training for CYBATHLON offered the perfect context to follow a user-centered approach, as presented in this work. Here, we present the evolution of our system, the implementation of two control alternatives, their selection, and the development process from previous events (i.e. *Cybathlon 2016*, *Cybathlon Rehacare 2018*, *Cybathlon Series 2019*) to the *Cybathlon 2020 Global Edition* race (please refer to Fig. 1 for the complete timeline). In addition, we present the performance at each event and our personal experience as a team. This work proves the versatility of the system towards the user’s preferences and the context demands. Finally, we discuss insights and future perspectives that arose from our CYBATHLON experience and training.

## The SoftHand Pro platform

**Fig. 2:**
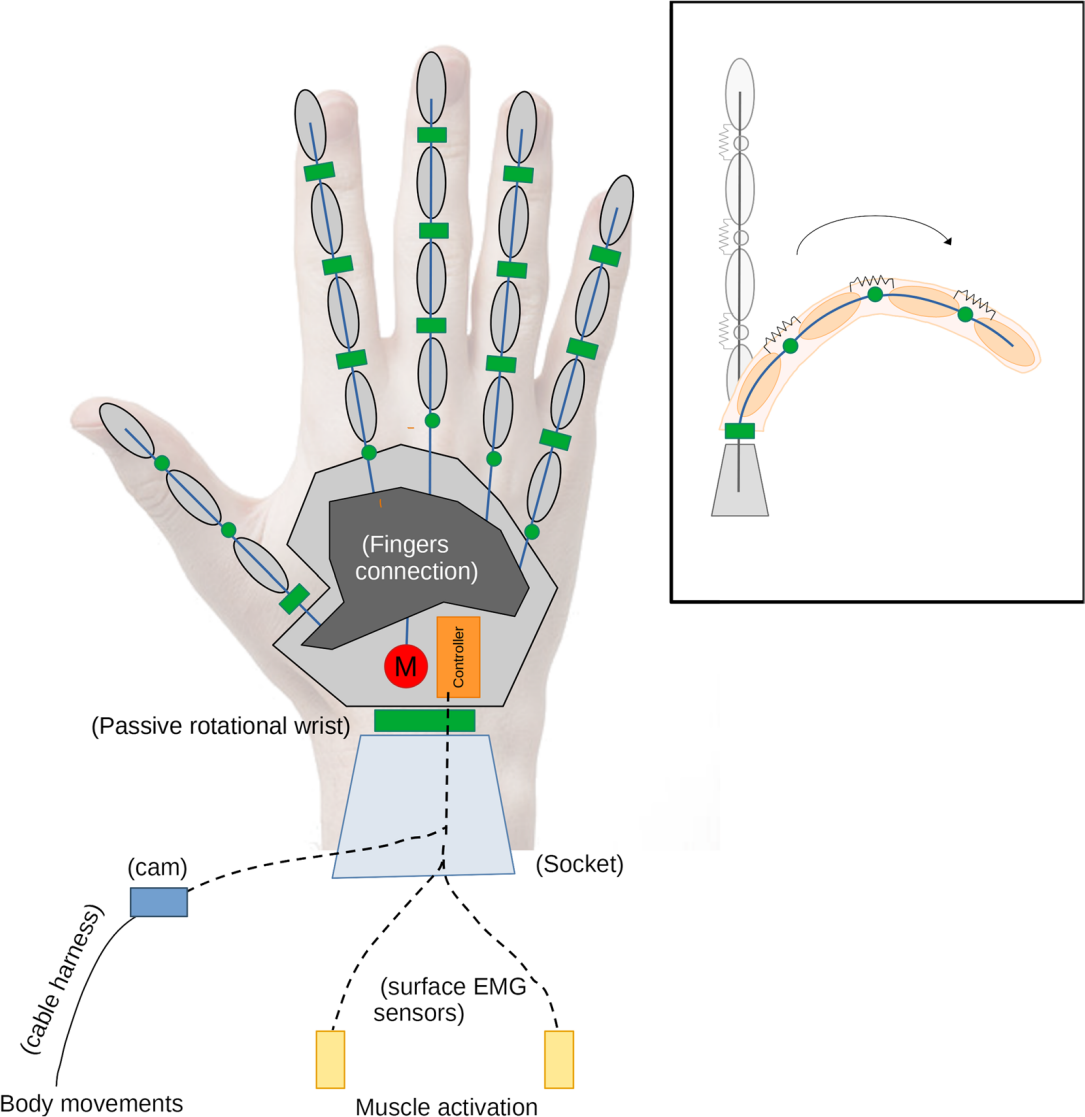
Concept of the SoftHand Pro platform. This solution combines the advantages of poly-articulated hands with a single control input, which may be actuated though body movements or muscle activation

The SoftHand Pro (SHP) implements one synergy thanks to the unique tendon (fingers blue lines and the fingers connection at the palm in Fig. 2) that simultaneously actuates all 19 joints (all green cylinders in Fig. 2, except for the passive rotational wrist). This architecture ensures a simplified control interface since the user must actively control only one degree-of-actuation while presenting the advantages of a poly-articulated hand. The specific soft joint mechanism of the hand makes possible the adaptation of its fingers to the object shape during the grasping action, ensuring a reliable grasp while commanding a unique open-closure pose (i.e. only one input to the controller in Fig. 2) and selecting an appropriate approach during the reaching phase.

Embedded intelligent machines present autonomous character that is not placed in the control architecture exclusively, but rather on the balance between morphology, materials and control [8]. This principle is exploited in the SHP through a differential mechanism used for its actuation, in combination with its soft-synergistic behavior (find additional details in [9]). This is an interesting feature in prosthetics, not only for the similarity to human hands but especially because of its control simplicity. This permits larger contract areas of its fingers and the “decision” of several grasping geometries when the contact occurs. Consequently, only when these novel features are exploited directly by people with limb deficiency, it is possible to validate the benefits of the embedded intelligence and potential of a flexible user interface platform. In our experience, participating in CYBATHLON has helped to integrate the user requirements in the SoftHand Pro platform development.

According to the SHP control simplicity, the control input can come from two alternatives (visible in Fig. 2) and a user-centered approach was used for its selection. One possible solution consists of a hybrid body-controlled version [10], where body movements are transmitted through a cable harness to a cam that electrically sends its position to the controller to be transmitted to the motor and proportionally commands the hand closure. This is an adequate alternative that permits body-control with less effort from the proximal joints. The alternative is a direct myoelectric control using two surface EMG sensors embedded in the socket and located at two agonist-antagonist muscles (forearm extensor and flexor), to open and close the device.

While the hybrid body-powered control method was specifically designed for *Cybathlon 2016*, the myoelectric alternative employs a standard direct control for a 1 DoF system. Proportional Velocity Control was used to command the SHP and hold position when the muscles are at rest. A First Come, First Served (FCFS; reported in [11]) approach was used to decide the signal that is sent as input to the device. This takes into consideration of a settled activation threshold value to detect intention of movement. A preliminary calibration of the method was done according to the muscle activity of each pilot, in order to find the most appropriate parameters.

## SoftHand Pro at Cybathlon 2016

The SoftHand Pro Team participated in *Cybathlon 2016* with a pilot (Pilot A, male) that presents a unilateral (right) transradial amputation. He lost his arm at the age of 14 and was left-hand dominant before amputation. The subject is a body-powered hook prosthesis user in his daily life. He participated in CYBATHLON when he was 29 years old and performed a rehearsal two years before the competition. The participant tried both the myoelectric and the body-controlled version of the SHP platform during the rehearsal and training, achieving good performance with both control methods. He decided to participate with the body-controlled option in *Cybathlon 2016* for comfort and confidence reasons. The combination of the SHP mechatronic design with a body-controlled actuation, developed following the user preferences and feedback, was called SoftHand Pro-H. For more details on its design process, please refer to [10, 12].

**Fig. 3:**
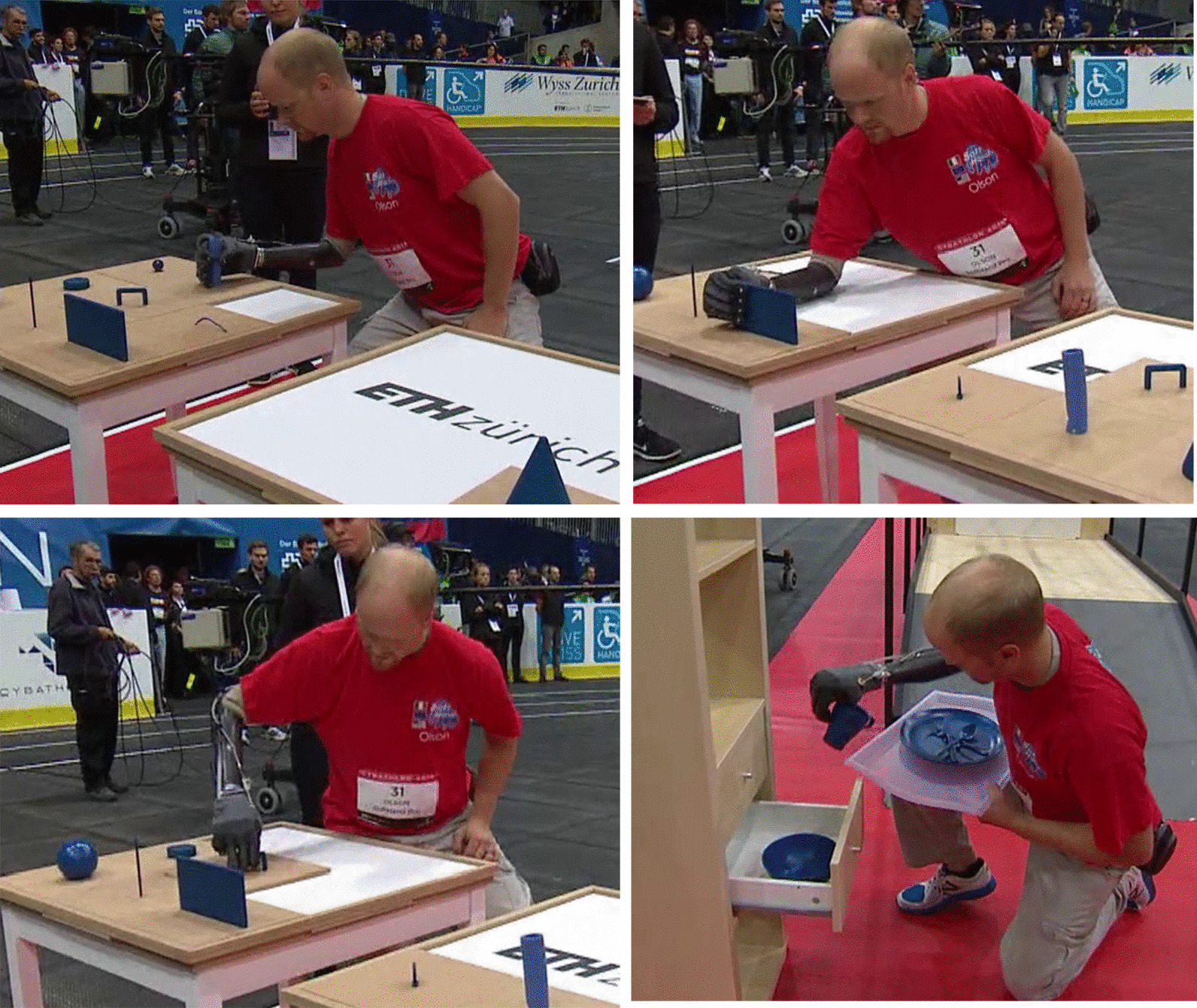
Multiple grasp behaviors observed in *Cybathlon 2016* during the grasp of different “blue objects”, that can be touched only with the prosthesis. These pictures refer to Task #1 and #3

**Fig. 4:**
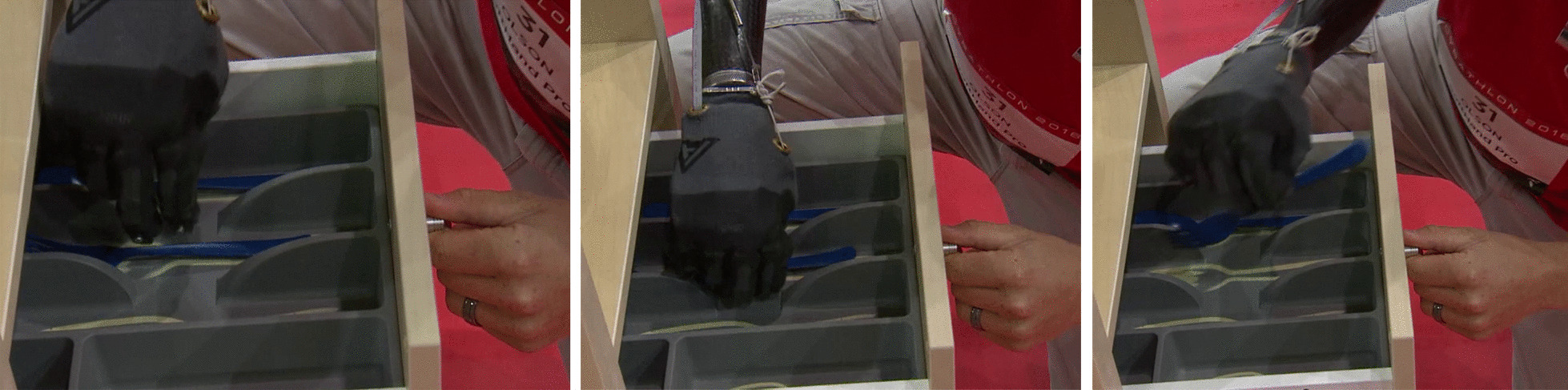
Photo-sequence of grasping a knife inside of a cutlery tray placed in a drawer during Task #3, Cybathlon 2016

*Cybathlon 2016* included tasks that require the manipulation of objects of various sizes and shapes to favor the use of multiple grips. It also evaluates the use of a wrist joint and the execution of compensatory movements, including the grasp of objects from different locations and heights. Please refer to Appendix A for a detailed description of the tasks of *Cybathlon 2016* competition. Figure 3 shows examples of different grasps, where the user explores both the differential mechanism in the SHP actuation and the selection of the most appropriate approach. Some grasps present contact with the palm, while others use the opposition of the fingertips (i.e. tripod grip). The photo-sequence of Fig. 4 presents the pilot grasping a knife inside a cutlery tray (i.e. narrow space), showing an intentional pre-grasping posture of the hand to favor a precise and more reliable grasp with its fingertips.

**Fig. 5:**
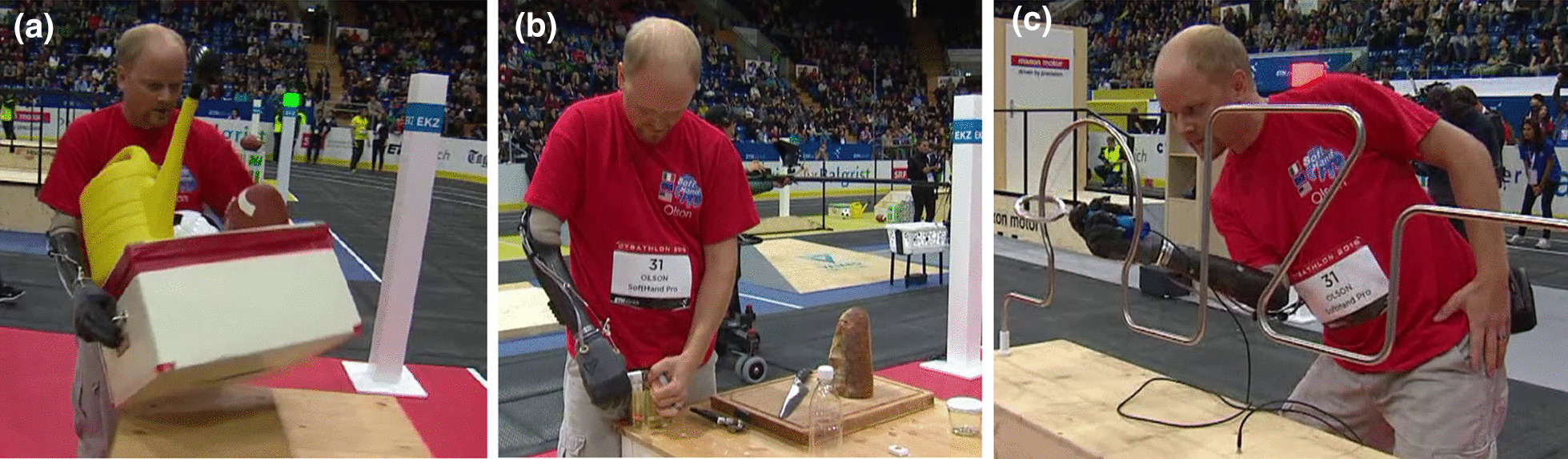
Specific improvements through the use of soft robotics and a body-controlled method. **a**, **b** showed natural body posture during bimanual activities, and **c** a reliable grasp even though the execution of large compensatory movements

The softness and adaptability of our technological solution favors natural body postures. This advantage is especially visible when coordinating both arms, as in Fig. 5a and b. The body-controlled modality permitted reliable grasps in those situations that, due to the lack of an active rotational wrist, large compensatory movements occurred, as in Fig. 5c. Large compensatory movements may compromise skin contact with EMG sensors in myoelectric solutions, or result in involuntary muscle activation.

Table 1 reports the results of our system in comparison with the winner of *Cybathlon 2016* and the average time per task (that considers all the participating teams that successfully completed the task). Only two teams could complete all the tasks of the CYBATHLON without penalty, the DIPO Power team, who won the event, and the SHP Team. DIPO Power team participated with a classic body-powered hook and was faster in all tasks except for tasks #2 and #3, which involve the movement of more proximal joints to grasp or manipulate in different locations or body configurations. Compared to the average timing per task, we may improve our performance for tasks #4 and #6. Additional results of this event are reported in [12].

**Table 1:** Cybathlon 2016—Time for each task achieved by DIPO Power (winner of the competition) and SoftHand Pro team, in comparison with the rest of participants

Rank	Time						Team
1	28	60	133	47	54	40	DIPO Power
5	42	56	132	66	66	41	SoftHand Pro
Av. Time	50.29	148	138	61.75	81.86	39.33	All teams
Task:	#1	#2	#3	#4	#5	#6	
Points:	115	102	130	104	108	101	

Overall, our participation in *Cybathlon 2016* was very satisfactory and highlighted the potential of this research prototype, even in comparison with several well-established commercially-available hands. The positive outcome of this experience showed that a simple yet dexterous solution with adaptable features could favor the grip of many objects, even in different locations, and gave us important insights on how to improve the system.

### Lessons learned from 2016

Forced configurations such as those created by restricting interaction surfaces represent a strong limitation in prosthetics. These can be attributed to (1) the specific size, geometry or properties of the object, (2) the location in which it is placed, and (3) its surrounding environment. In the CYBATHLON race, these conditions are recreated by objects with blue surfaces, which can be only grasped with prostheses.

**Fig. 6:**
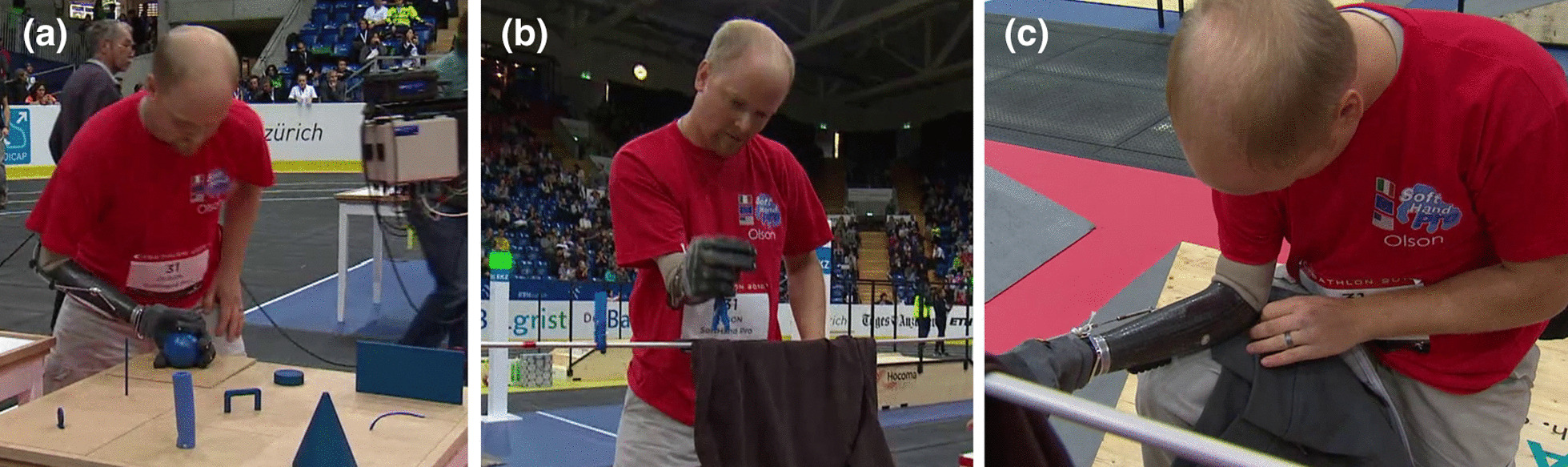
Examples of aspects to be improved. **a**–**c** Refer to the precision grasp

Figure 6a demonstrates how, due to the presence of blue objects, unnatural human grasps may be explored with the SHP. Although this can be a practical feature in the context of a competition, many users in everyday situations might not accept this. Furthermore, the strongest grasp of the SHP is achieved by applying force with the fingers against the palmar area. For instance, the blue clothespins were grasped in this manner (see Fig. 6b), instead of exploiting the contact between the index and thumb fingertips. Note that poor precise grasps may jeopardize the active use of the prosthesis in unconstrained conditions, as occurs in Fig. 6c. Accordingly, the improvement of precise grasping of small objects is our highest priority modification for future events.

Furthermore, prosthesis users often avoid using their robotic hands for interaction with other people and use them exclusively for manipulation purposes. However, the compliance of our prosthetic hand could permit soft interaction with oneself and with others. Due to the importance of this aspect in prostheses acceptance, a second priority is the refinement of the system for a less industrial design to break social barriers and permit a higher perception of safety.

## System developments

### Hardware features

**Fig. 7:**
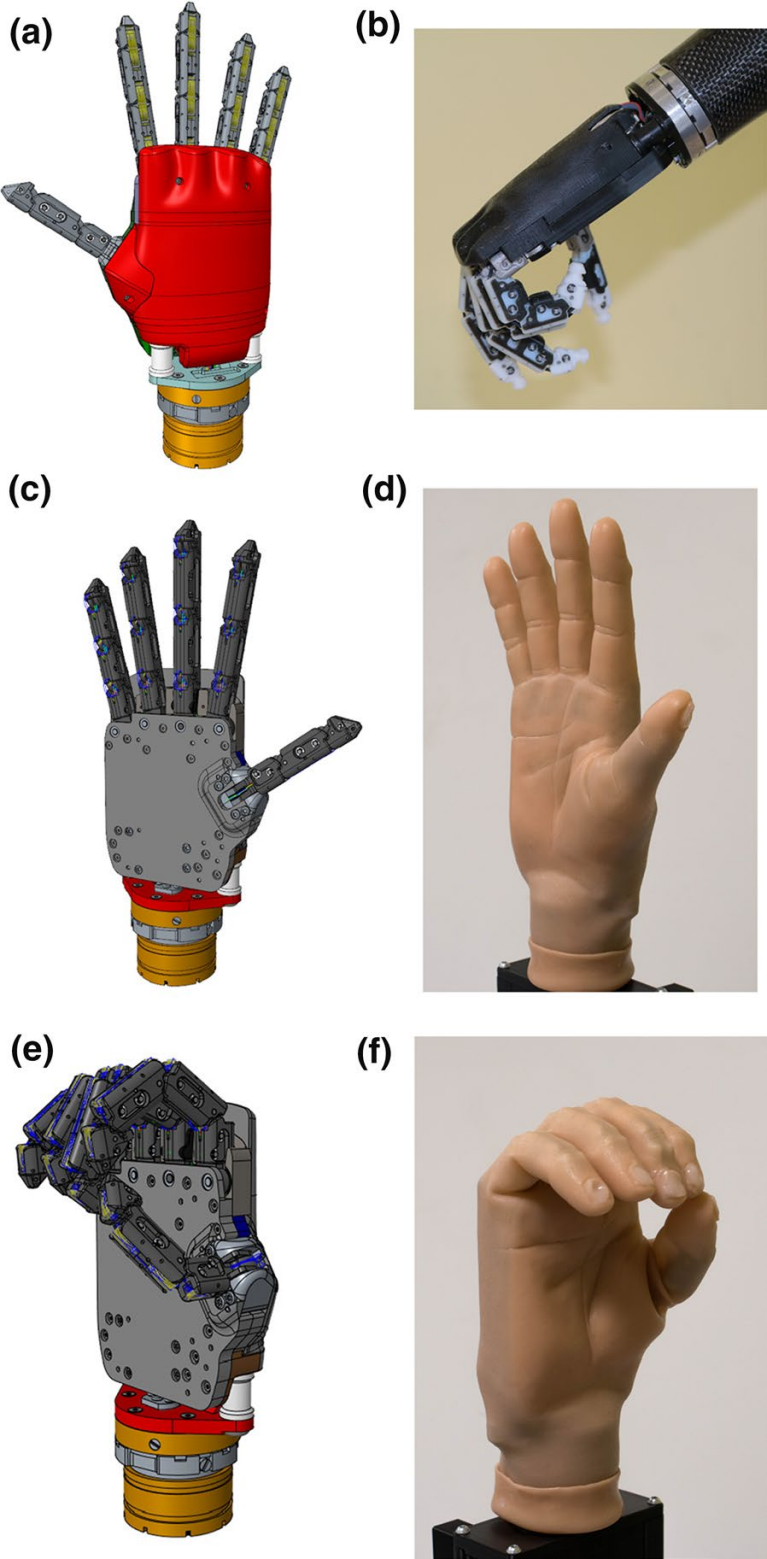
Novel version of the SoftHand Pro. **a** Shows the CAD model and **b** the manufactured components mounted on a socket. **c**–**f** Present different views of the new version of the SHP with a skin-toned glove

Several actions have been taken to improve the mechanical design of the SHP platform. Figure 7a shows an isometric CAD model of the hand+wrist components of the SHP platform. Figure 7b presents the prototype connected to the socket module of a patient and at a certain level of closure to show the interconnection among phalanges. Please, refer to Fig. 7c–f for a clear view of two positions of the prosthesis (hand open and partially closed), and their corresponding CAD model.

(i) *Weight and size:* An important aim of design development was reducing the weight and size of the device, as this is a source of discomfort and a common reason for the abandonment of upper-limb prostheses. By reducing the SHP palm circumference, fingers thickness and the overall weight, the hand’s size (from 210.82 to 177.8 mm—measured from the tip of the longest finger to the limit of the palm) and weight (from 520 to 290 g, SHP + Quick disconnect wrist from Ottobock) more closely resemble those of an average adult female (172.72 mm) and male hand (193.04 mm). The current hand prototype opens a new perspective in prosthetics literature and provides a novel area of research (e.g. [13]).
(ii) *Actuation system:* The SHP control of this version combines a DC motor with an epicicloidal gearbox with a custom-designed worm gear. This design feature resulted in a reduction of the overall dimensions of the actuation system, an increase in the torque performance of the actuation unit, and a reduction in energy consumption because of its non-back-drivability (during the holding phases, the DC motor is not active and no current is absorbed), thereby increasing battery life. This solution allows the user to turn off the hand and maintain the grasp during a long holding phase, reducing the risk of unintentional activation/opening of the hand when holding an object for a long time, which is especially interesting when using the myoelectric control modality.
(iii) *Grip force:* The first version of the SHP exerts a maximum grasping force of 26 and 76 N during precision (pinch) and power grasps, respectively, and sustains a maximum load of 100 N. Likewise, this SHP version can exert a maximum 45 and 88 N for precision (tripod) and power grasps, respectively, and can sustain 130 N. Force characteristics are critically important for tasks involving the use of heavy tools and carrying heavy bags, common activities of daily living.
(iv) *Durability:* Hand prostheses are exposed to intensive use in performing a wide variety of activities. Therefore, an important factor is the system durability, i.e., the ability of the prosthetic hand to withstand wear, pressure, or damage of its components caused by a large number of mechanical cycles (opening/closing) without breaking. The SHP platform maintained its mechanical life being capable of 494,000 cycles before the tendon breaks, which corresponds to an average use of a myoelectric hand for 3 years [14]. Similarly, the battery life is a very important factor as it allows longer uninterrupted use. The battery autonomy went from 3 to 10 h.
(v) *Precision grasp:* An aspect of special interest concerns the final position of fingertips after closure, which we tried to achieve a more precise grasp. Previously, the thumb tip made contact with the second phalanges of the index and middle fingers, hampering the pinch performance. By increasing the elasticity of the bands connecting the phalanges, the ring and little fingers move after a short delay with respect to the thumb, index and middle fingers, thus avoiding unwanted contact of the ring and little fingers with small objects. The favor of a tripod grasp closure (instead of a pure pinch) permits a more stable grasp of small objects. In the case of thin or flat shapes, the tripod grasp closure also allows for varied positioning of the object between the fingers to spread the contact forces. Although Fig. 7e and f highlight the pinch-tripod closure, a fully closed configuration of the hand is still possible to achieve a power grasp. In addition, the SHP platform presents a passive extension wrist that allows elastic deformations of the hand orientation for a more natural body position when needed.
(vi) *Aesthetics:* The cosmetic appearance of hand prostheses is an important factor that users take into consideration when adopting a prosthetic system. The current version of the SHP can be used not only with commercial working gloves (as in *Cybathlon 2016*), but also with a cosmetic silicone glove (skin-toned in Fig. 7d and f). The glove was customized to match the mechanical features of the SHP, thus improving the hand cosmetics and providing an additional layer of elasticity. The silicone glove becomes a fundamental part of the hardware as it defines the final tuning of the elastic components (i.e. between phalanges) of our hand to define precisely its closure and aperture trajectories. Moreover, this glove increases the grip traction on the overall hand and presents a better fit of the hand, i.e with no internal displacement between parts, to obtain a higher precision when manipulating objects.

### Software features

The novel version of the SHP platform has on-board firmware that can record variables associated with the quantity and type of daily usage of the hand. This permits the later analysis of its use, which is especially useful in long-term studies. Moreover, a graphical user interface (GUI) helps prosthetists and researchers with the use and setup of the hand, respectively. The GUI provides easy access to configuration parameters (e.g., maximum current values, EMG signals), data stored in the hand memory, and log of the internal status of the hand, e.g., power consumption, reading of position encoders.

This version retains the original features described in Fig. 2, including the possibility to use both control modalities. Regarding the myoelectric control modality, the SHP firmware offers the flexibility of using one or two surface EMG inputs. If necessary, the SHP can be configured so that it is passively driven to the fully-closed or -open state, and then only one EMG channel is used for actuation. This option is particularly useful for subjects who have difficulties or are unable to generate two independent myoelectric input signals. Otherwise, we used FCFS with two sensors, which increases the control robustness, or the the hybrid body-powered control solution used for *Cybathlon 2016* [10] as an alternative.

## In-preparation CYBATHLON events

To prepare for *Cybathlon 2020 Global Edition*, the SoftHand Pro Team has trained with two pilots (both females). Pilot B has a congenital unilateral (left) limb deficiency at the transradial level. The subject uses a cosmetic prosthesis in her daily life, but was familiar with myoelectric control. She participated in *Cybathlon 2020 Global Edition* when she was 40 years old and performed some rehearsal runs and participated in other in-preparation CYBATHLON events (see Fig. 1). Pilot C presents a transradial amputation on the left arm at a very young age. She participated in Cybathlon Series 2019 when she was 23 years old and performed some rehearsal runs with no experience in either myoelectric or body-powered control, as she usually does not wear any prostheses. Similar to Pilot A, the participants initially tried both the myoelectric and the body-controlled versions and finally decided to participate with the myoelectric option in the races. Table 2 shows a summary of the comparison for two outcome measures (the Assessment of Capacity for Myoelectric Control (ACMC) and the System Usability Scale (SUS)) with three configurations: myoelectric prosthesis (MP), body-controlled (BC) with two configurations (indicated as -c1 and -c2).

**Table 2:** Analysis of the control modalities and users’ preferences in preparation for *Cybathlon 2020 Global Edition* [data reported in [15]

	Pilot B			Pilot C		
	MP	BC-c1	BC-c2	MP	BC-c1	BC-c2
ACMC	55.4	56.3	51.9	53.7	50.3	47
SUS	90	52.5	37.5	85	75	62.5

* MP* myoelectric prosthesis,* BC* body-controlled ($c_i$ = configuration).* ACMC* Assessment of Capacity for Myoelectric Control,* SUS* System Usability Scale

In both cases, although functional performance is quite similar and in the same clinical scale range (i.e. generally capable) for the ACMC, the SUS score highlights their preference for the myoelectric control alternative (with a score of 90 and 85 for Pilot B and C, respectively). These outcome measures were intended for CYBATHLON, but they were used for another research study that includes an additional patient [15]. Considering that the sensibility of both outcome measures is similar, while results from the functional assessment (i.e. ACMC) are influenced by both the mechanical features and the control method performance, the main effect in the user-perception survey (i.e. SUS) depends exclusively on the control method, as the perception of hardware remained constant. Results suggested that the properties of the physical device contribute to the overall performance with a higher percentage. However, the suitability of the control method is fundamental for the final acceptance of the prosthesis. For this reason, the myoelectric control modality was the selected solution for both pilots.

**Fig. 8:**
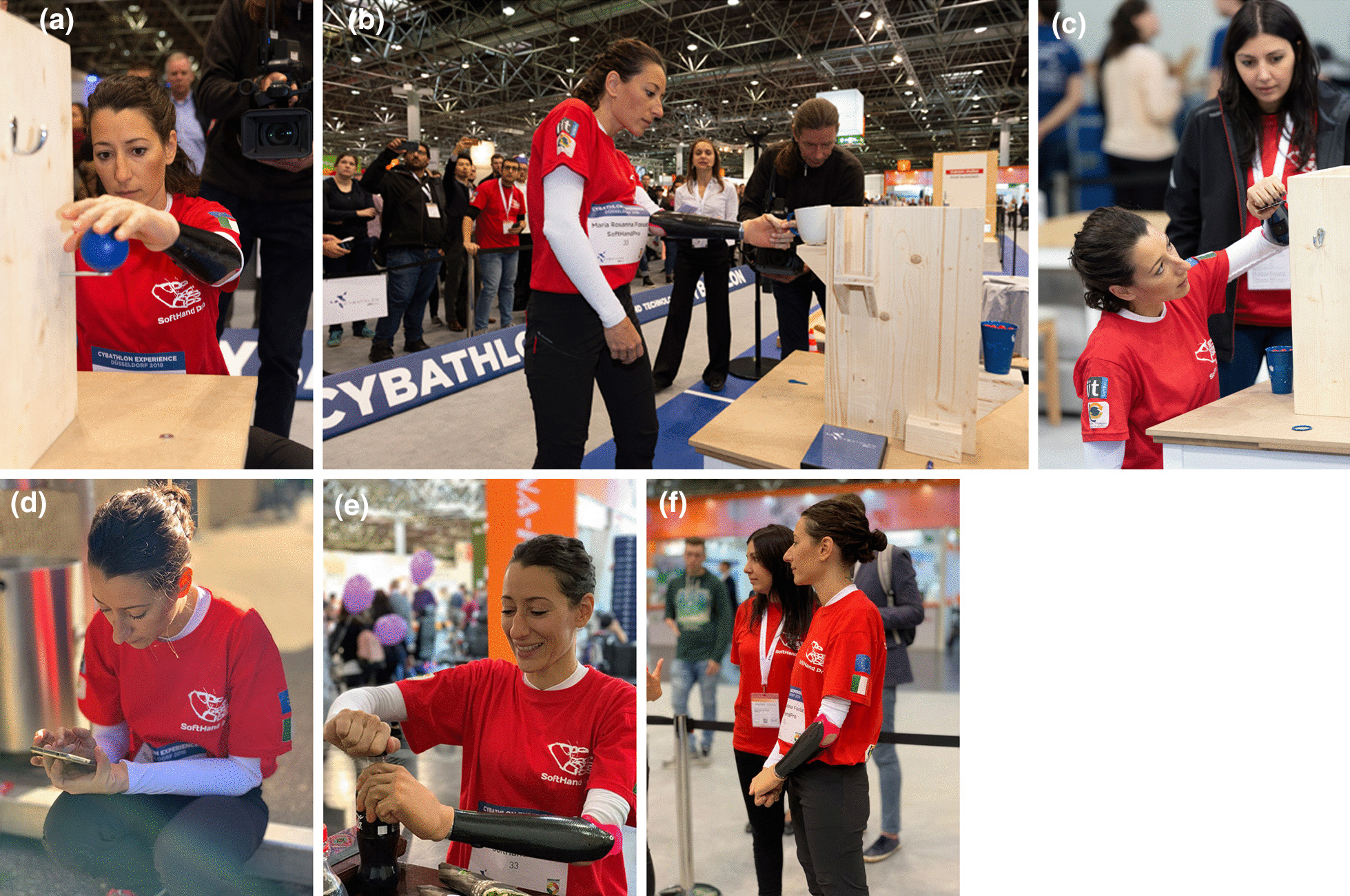
SoftHand Pro at *Cybathlon Rehacare 2018*: **a**–**c** present examples of the Pilot B executing some of the novel tasks with the developed version of the SHP. **d**–**f** Shows examples of additional activities (soft interaction and ADL)

**Fig. 9:**
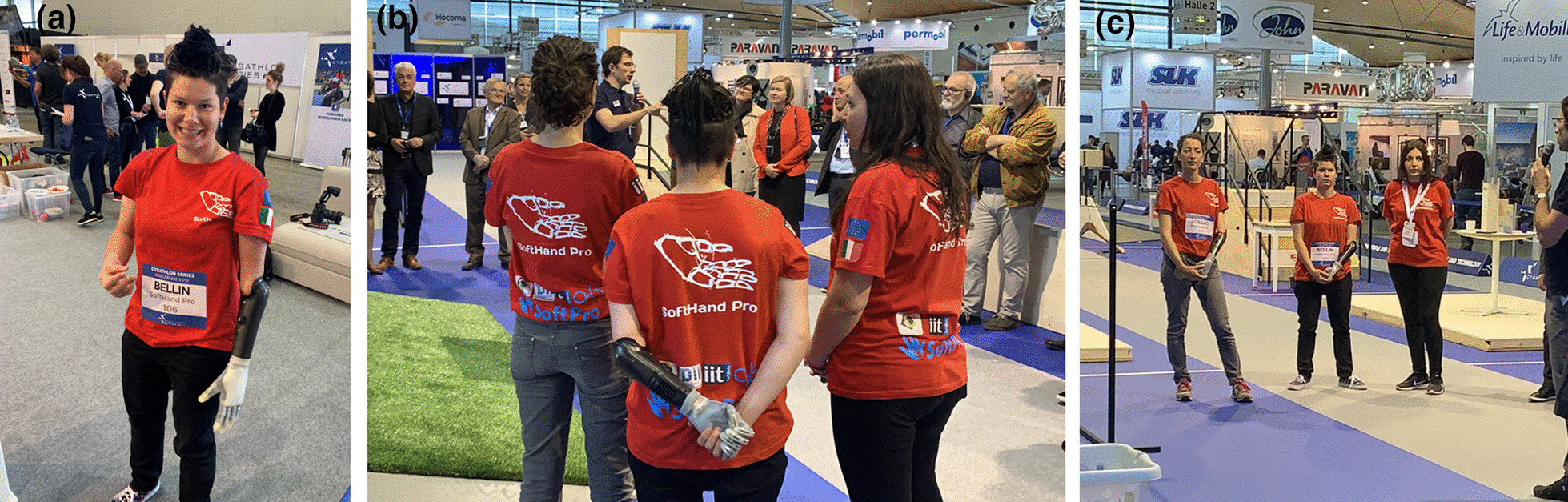
SoftHand Pro at Cybathlon Series 2019: **a** Pilot C testing our system with the transparent glove, **b** highlights the soft interaction with her controlateral limb, which is repeated in **c** by both Pilots showing a natural posture while waiting in the race

Figures 8 and 9 show examples of both *Cybathlon Rehacare 2018* and *Cybathlon Series 2019*, two in-preparation events in which we participated. Especially interesting are Fig. 8e–f, that present examples of functionality and natural hand shapes spontaneously achieved before and after the race. This bimanual coordination and active use suggest a certain level of embodiment of the device favored by the built-in softness and its resemblance to the human hand function and structure. A more extensive investigation is proposed in [13], which show a quantitative evaluation conducted during a clinical trial.

**Fig. 10:**
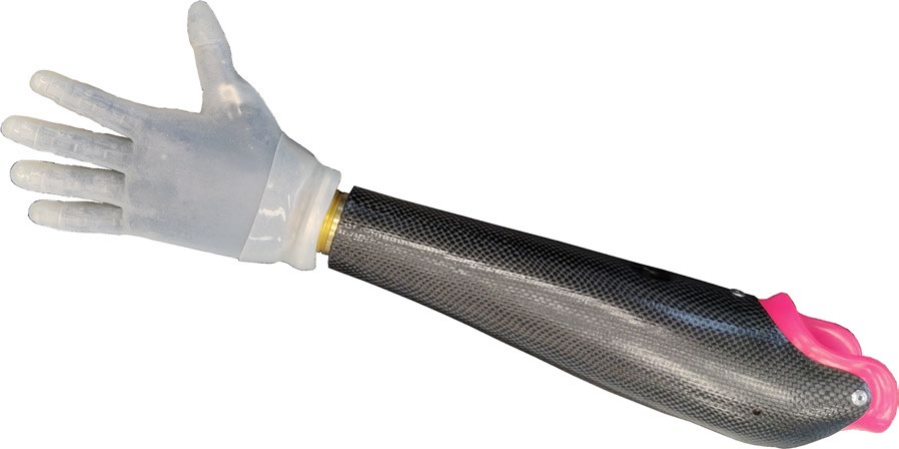
Complete system that participated in Cybathlon 2020 Global Edition

Even though Pilot B used the skin-toned glove in 2018, the subject asked for the creation of a no skin-toned and without nails glove (in Fig. 10), for a less human-like alternative. While human-like appearance has been always strongly appreciated in prostheses, state-of-art modern devices evidence an emerging tendency toward more machine-like aesthetics. The *uncanny valley* is a term used to describe the relationship between the human-like appearance of a robotic object and the emotional response it evokes [16]. Although robots can be extremely realistic and lifelike, usually when we examine them, they present important differences from the human template. According to this phenomenon, people feel a sense of unease or discomfort in response to humanoid robots that are highly realistic but lack functionality. If this is the case, devices that are made to mimic the human touch may end up alienating people using such tools, which is a dramatic effect in the case of prosthesis users. Therefore, a more robotic but still appealing glove represents a more adequate option for many subjects.

Figure 9a shows Pilot C during the *Cybathlon Series 2019*, who also chose the transparent glove for the SHP. Figure 9b and c present additional examples of natural postures spontaneously achieved by both Pilots before and after the competition. All the technical changes showed an improvement during the in-preparation event (*Cybathlon Series 2019*), where Pilot B scored 2nd and Pilot C 4th.

## Training for Cybathlon 2020 Global Edition: the race and soft features inclusion

The participation to CYBATHLON always requires a certain degree of involvement both from the pilots and the technical team to prepare the system and explore its capabilities for its best performance. The selection process of the control method was before the training sessions, but once that the training started, the control modality remained constant. Note that the training also helped the team in selecting the definitive control thresholds and the specific level of stiffness in the elastics for the execution of all tasks. Furthermore, it allowed us to evaluate the control robustness in different arm locations and the durability of tendons and other mechatronics components. Accordingly, the training here refers to the pilot learning to use the system or finding the most convenient manner to perform a task without changing the control modality. The soft properties of our system, which are not yet available in the market, represent an additional component to be trained in order to properly explore and integrate this novel feature. The final design, including the socket module (with the inner socket), with which Pilot B participated in *Cybathlon 2020 Global Edition* is visible in Fig. 10.

The training regimen consisted of several 2h-sessions for 3 months. The duration was decided not to increase excessively fatigue of the pilot, but to improve the pilot’s competence and familiarity with the race. The frequency of these sessions increased gradually from one day per week to a dual session (morning and afternoon) every day during the 2 weeks before the competition. The event was recreated in the lab with different setups. Even though some of the real objects were not available until the last part of the training, most of the recreated setups and objects were similar enough to increase the confidence of the pilot in the real competition.

**Fig. 11:**
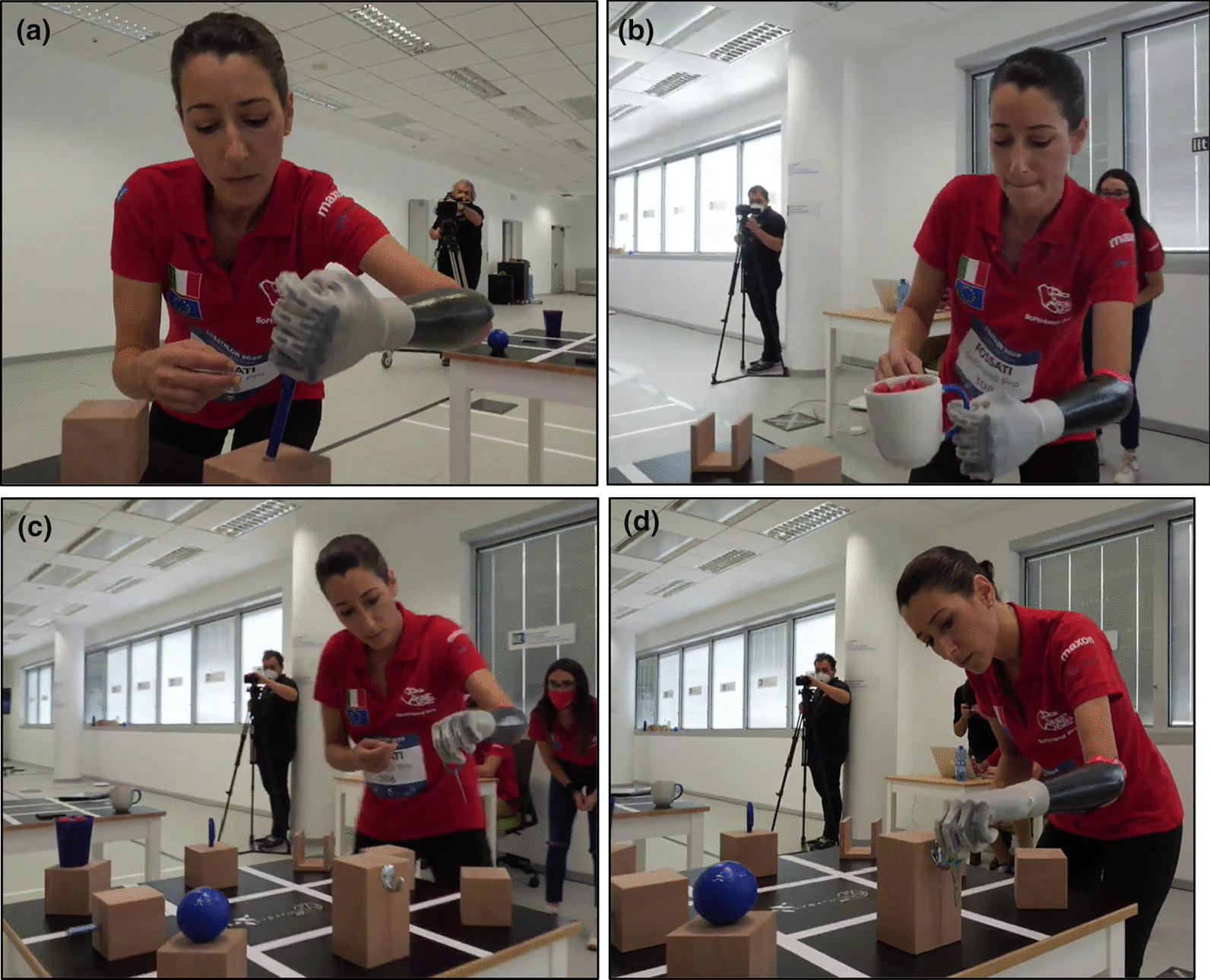
Different grasping patterns corresponding to the object shape and requirements: **a** a pen, **b** a mug full of wooden-balls and **c**, **d** are different instants of the grasping and moving of a key

Initially, we trained the grasping of the different objects and the execution of actions included in each task. Figure 11 shows some of the examples of different grasps, from more powerful to precise, depending on the properties and shape of the object required. Then, we trained the time needed for the completion of the whole task. Finally, we also prepared simulations of the whole race. This process allowed first to improve the performance of each part included, and then, test the competition conditions. At the latter stage, we also trained the worst-case scenarios where we forced objects to fall down during the race so that the pilot must try to grab them in non-ideal conditions and/or continue with the race without those points and wasting time.

**Fig. 12:**
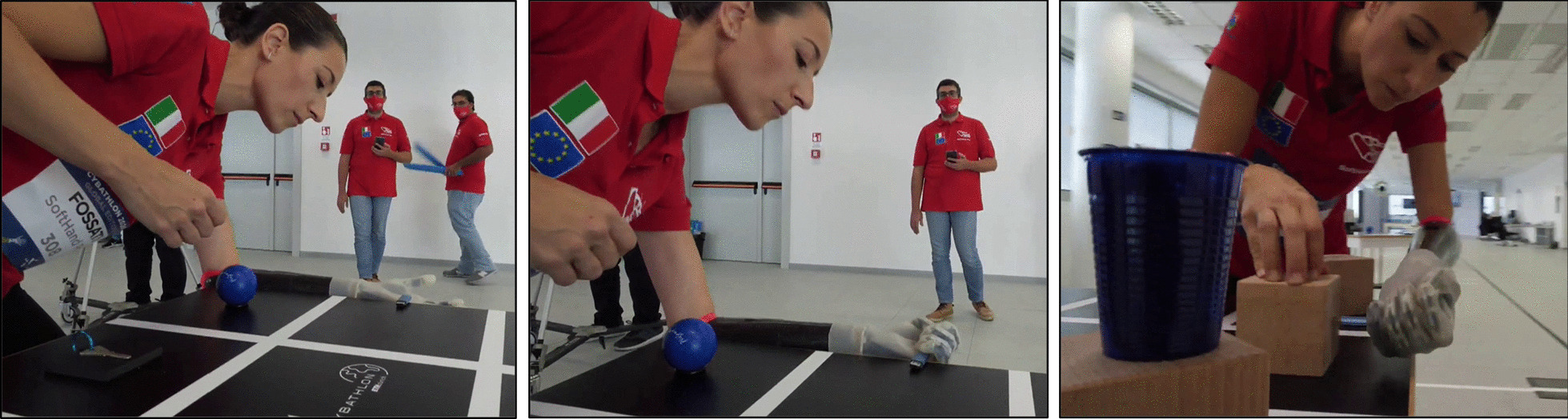
Photo-sequence for the grasping of a USB pendrive: The object requires a certain approach for a safe grasping, holding and releasing. Tasks that requires challenging release are fundamental to explore more difficult grasping actions

The race evaluates not only the grasping of an object, which is the standard method to assess the capabilities of a prosthesis, but also the reaching, holding and releasing phases of the action, which are fundamental for a true inclusion of the prosthesis in daily life. Goal-oriented actions sometimes force a different grasping pattern as the completion of the goal (i.e. at the release phase) is more important than the grasping. Figure 12 shows an example of that, in which the precision required to introduce a USB pendrive forces the SoftHand Pro to explore a less common grasp pattern to be able to grasp, hold it and release it properly. Therefore, the training sessions influenced the functional abilities of the pilot to use the prosthesis, be confident, also in precise controlling the system movement, and able to complete all actions in a good time. Functional improvements were observed by the technical members of the team though lower execution times, reduction of grasping failure, higher active use of the prosthesis during the race and higher independence (i.e. less support from the training team) during the race simulations.

## Cybathlon 2020 experience

**Fig. 13:**
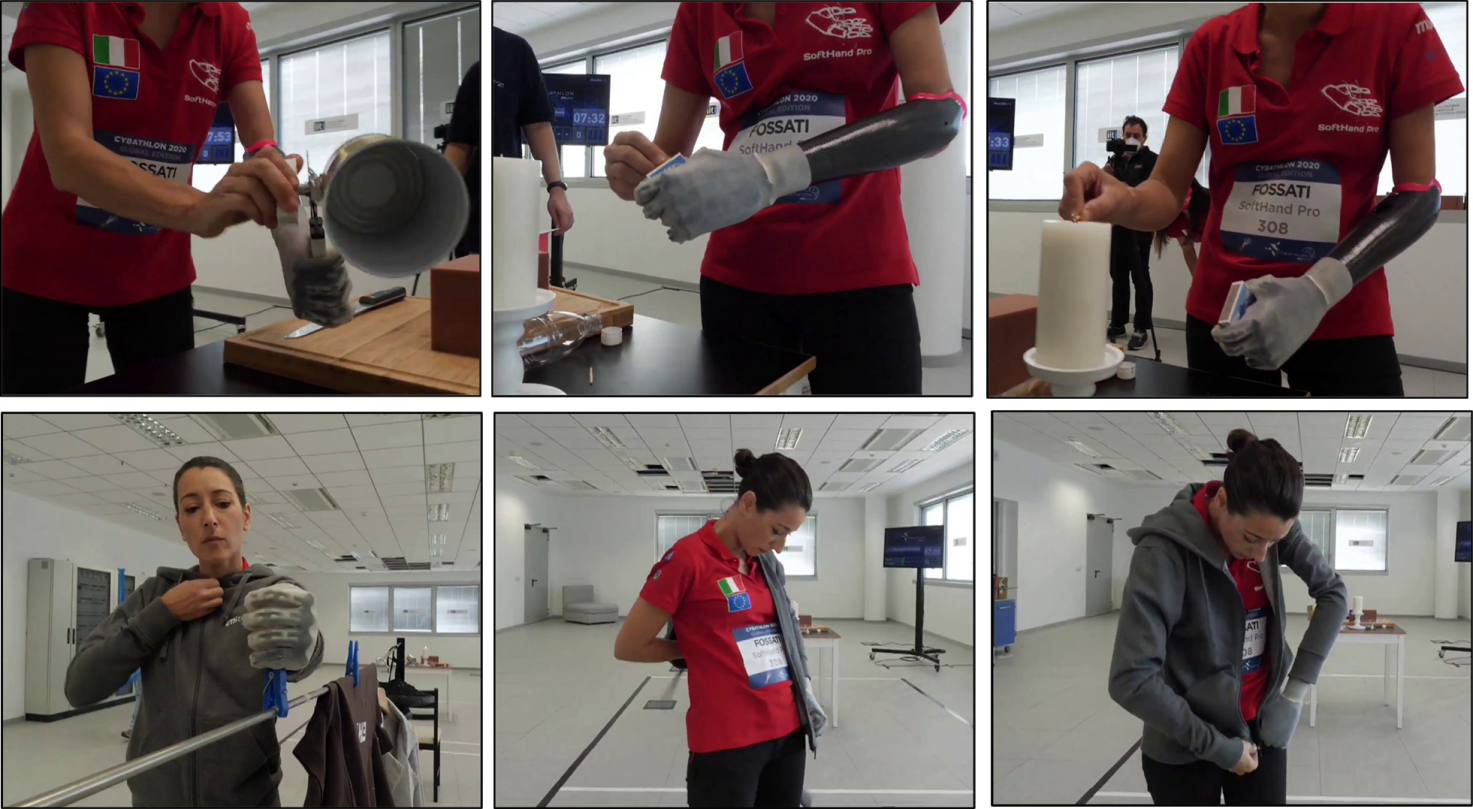
Bimanual activities and multi-tasking: examples where both hands are used and/or needed for the same action to improve the performance

Figure 10 shows the complete system that participated in *Cybathlon 2020 Global Edition* with Pilot B in the myoelectric control modality. Please refer to Appendix B for a detailed description of the tasks of this competition. The results from our experience in the competition were good: our team won the silver medal of the competition, but also Pilot B completed all tasks on time and the SHP resulted as the fastest myoelectrically controlled prosthesis. Table 3 shows the most significant results of the competition for our system, in comparison with the winner, the Maker Hand team. Comparing the two first rows, we observed a faster execution of bimanual tasks for the SHP (based on Task #1 and #2, which includes standard Activities of Daily Living (ADL) that favor the coordination of both hands). Figure 13 presents examples of the two tasks in which Pilot B showed bimanual manipulation and both arms coordination for proper execution and integration of the prosthesis.

**Table 3:** Cybathlon 2020 Global Edition—Time for each task achieved by Maker Hand (winner of the competition) and SoftHand Pro team, in comparison with the average time for all participants that succesfully complete the corresponding task

Rank	Time						Team
1	73	64	51	64	48	44	Maker Hand
*2*	*52*	*57*	*70*	*96*	*61*	*86*	*SoftHand Pro*
3	48	58	73	66	61	100	SoftHand Pro
10	42	63	90	70	0	97	SoftHand Pro
Av. SHP	47.33	59.33	77.67	77.33	61	94.33	SoftHand Pro
Av. Time	65.73	105.87	93.89	87.86	73.81	65.17	All teams
Task:	#1	#2	#3	#4	#5	#6	
Points:	14	15	16	17	20	18	

The race chosen by CYBATHLON among the three trials is given in italics

Rows 2, 3 and 4 show the results for all the three races done by our system before selecting the best one (considered as the final race for the competition). Observe that from all races, only Task #5 was missed for one of them, due to difficulties in sensory feedback.

Rows 5 and 6 present results of the average timing per task for both the SoftHand Pro (among the three races, only considering successful tasks) and all the participants to *Cybathlon 2020 Global Edition*. From here, we can observe in which task our system could improve compared to the average participating technology. Results point out the need for an improvement in Task #6, which is the only one with a longer duration than the average. Probably, this is influenced by the short residual limb of Pilot B and the use of a passive rotational wrist, whose configuration can not be modified once the object is grasped. In addition, the soft properties of the hand could disfavor a stable release in Task #6.

### User observations

We conducted an informal interview with Pilot B to get her feedback on the system used during the *Cybathlon 2020 Global Edition*. A less-desirable aspect of the system was its repeatability in grasping, which could be detrimental in a competitive scenario. Precise control of the grasping force, accuracy and geometry are important aspects which contribute to the repeatability of the grasp. Nonetheless, the increased adaptability that present soft-synergistic hands results in poor repeatability of its grasping geometry. Although she expressed her appreciation for the adaptability and compliance embedded in the unique design of the SHP, these features might have an adverse effect in a very structured competition. She pointed out that these are very convenient and excellent features in prostheses to perform multiple grasps and face many different tasks in everyday life environments. However, because the foremost goal in competitions is to perform the fastest, compliance may hinder the repetitive execution of the hand grasp during training sessions.

Nonetheless, the pilot found the grasp pattern on the SHP, which is defined by its compliance, very helpful, noting that it provided her with great confidence in the execution of all tasks included. The pilot was indeed pleasantly surprised by the dexterity of this hand, stating no need for a multi-grip commercial device. Further, she found the prosthesis weight and size match her expectations for a female version of the prosthesis, which also favors comfortability.

Concerning the challenges proposed by CYBATHLON, the new rules of the race emphasize the demand for an active rotational wrist. The pilot hopes that in the next years, research and technological development will provide wrist options with both functional and simple control solutions, suitable for those subjects with only a few signals available in the residual limb. She also appreciated that the race tasks included everyday life activities, such as putting on a sweater, for a proper acceptance of these technologies outside the lab or at home environments. She found this action important for social inclusion, and mentioned that further developments must sustain a smoother execution of the action.

On one hand, the specific rules of the CYBATHLON race may force specific grasp types or approaches that feel unnatural and result in non-optimal grips of the prosthesis. For example, the orientation of the hammer and the constraint of only being allowed to grasp it at certain points forced her to hold the object with a particular approach. In this configuration, the prosthesis could not provide a high level of force, and this may compromise the successful execution of the task. On the other hand, she recognized that the structured environment and strict rules had driven forward the capabilities of the prosthesis and its use in situations that the pilot would never have thought to be capable (e.g. cutting with the scissors), due to the common supporting role that prostheses present in unilateral subjects (Fig. 13).

## Lessons learned in 2020 and future developments

**Fig. 14:**
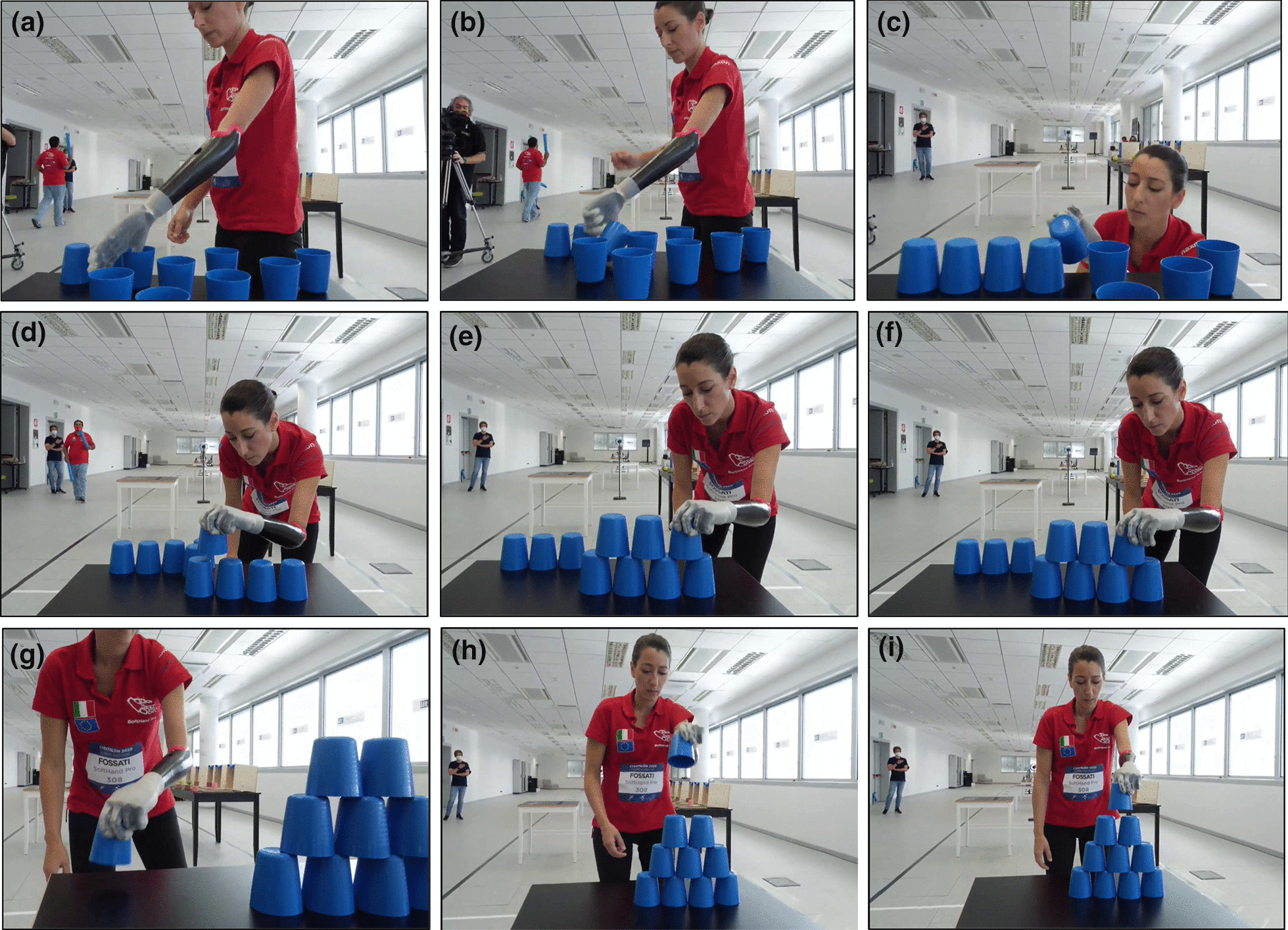
Photo-sequences of the stacking Task (#6, Cybathlon 2020). **a**–**c** Show the turning glasses phase. **d**–**f** Present the building of the pyramid, paying attention to the releasing due to the lack of sensory feedback and the instability of the pyramid. **g**–**i** Show the last glass positioning, which is the most difficult one and, because of the height of the Pilot B, it requires an unnatural position of the shoulder to release the glass parallel to floor

As observed in Table 3, the SoftHand Pro required larger execution times (both in a single track and average time) to complete Task #6, if compared with the average time (Av. Time) of the other participants. Task #6 is the one that mostly requires the use of a rotational wrist, as all the cups must be turned upside down to create a pyramid. The constraints added by the blue structures limited the exploration of the environment to perform a safe grasp. In addition, the reliability in the grasp while doing body movements and the level of accuracy in the releasing required by this task, led to a considerable amount of time used to avoid errors. Figure 14 shows a photo-sequence of this task. Future development will focus on improving this condition by possibly including an active rotational wrist. Note that this decision will require also a control method that can be intuitive for the pilot and does not compromise the hand movements, especially during the holding phase.

Soft synergies permit the exploration of external contacts to create several hand geometries or behaviors. This can be a feature but also a limitation, depending on the requirements. While this as a feature allows a very simple control with a higher adaptation to the objects or environment, providing several safe grasping actions, the embedded intelligence in its mechanics also hinders the repeatability of the hand grasp (i.e. larger uncertainty compared to rigid hands). Therefore, soft synergies require certain training to learn how to exploit softness and find the best grasping option possible in each condition (i.e. fast, reliable and safe grasping geometry). This requires additional work both from the pilot and technical team to investigate non-direct alternatives and find a desired and optimal grasping pattern for the race.

**Fig. 15:**
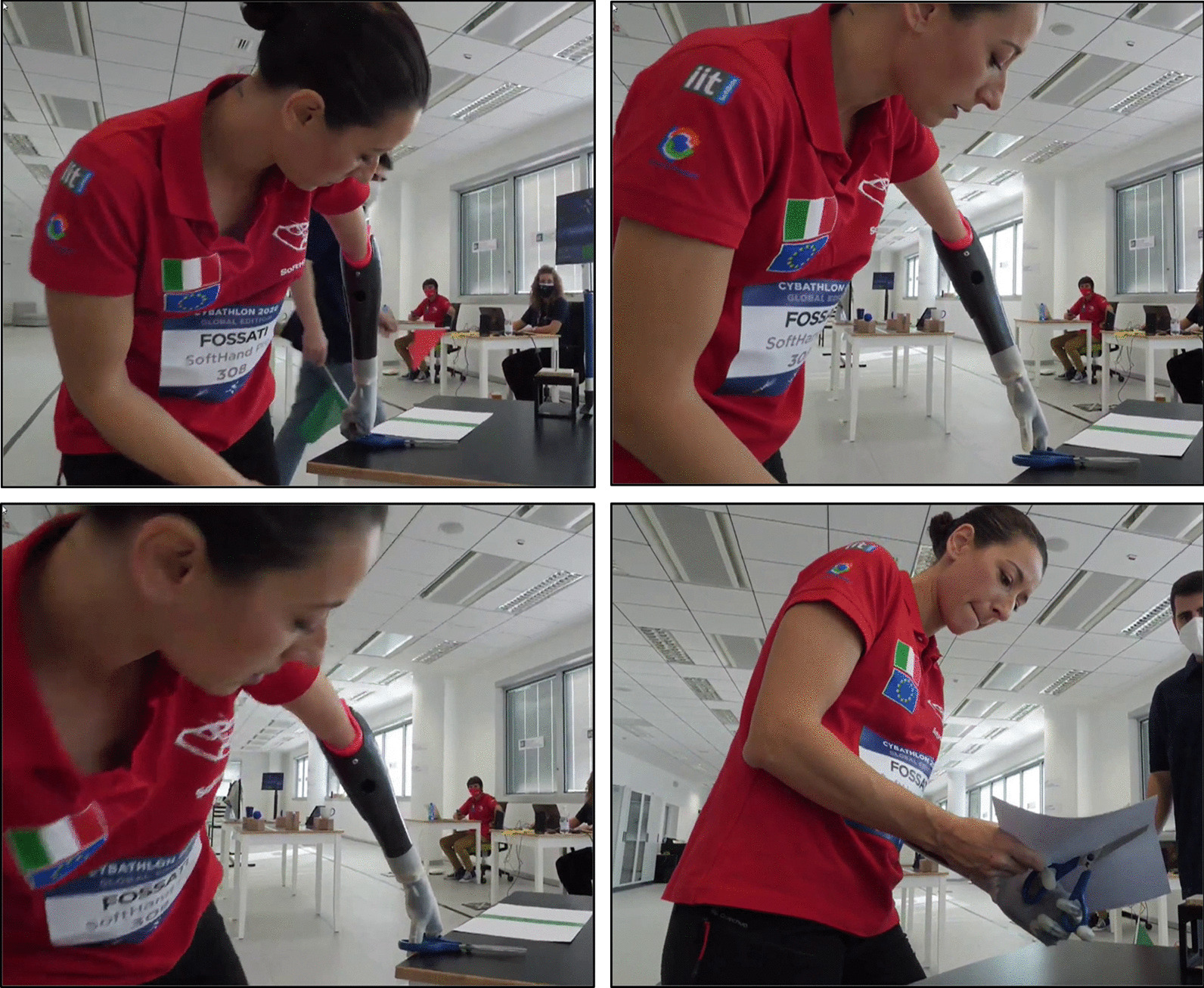
Photo-sequence for the grasping and use of the scissors (in Task #4, Cybathlon 2020): The strict requirement of touching the scissors only with the prosthesis may force an unnatural grasping approach or body posture for the success of the action

Especially for soft-synergistic systems, forced configurations, such as those created by blue surfaces represent a strong limitation, as the major feature of the SHP is the capability to explore the environment to execute different grasp patterns. Blue surfaces are only allowed to be contacted with the prosthesis, which in some cases jeopardizes the success of the grasp. Usually, the interaction of the contact forces between the hand and object favors the optimal grasp pattern for the SHP, and a slight object slip can compromise the adequate execution of the grasp. Sometimes this limitation forces our technology to find a non-natural way of grasping due to the lack of normal forces to adapt the object into the hand. Figure 15 shows an example of this situation.

In addition, Task #5 highlights the importance of sensory feedback in a prosthesis. Although the task only requires the recognition of simple object shapes and materials, which is simple information for human hands, it may be extremely difficult for prosthesis users, because of the lack of sensory feedback. Although visual feedback was not available during the task, pilots still rely on some feedback given by the exploration of the environment or the use of the hand (i.e. through the noise of the motors and/or the system). However, some objects are still difficult to distinguish, especially rigid objects. Soft objects can be discriminated against according to the level of compliance as some sort of force feedback is perceived by the user due to the rigid link to the residual limb muscles created by the socket. For rigid objects, due to the small changes on their corners, it was especially difficult for Pilot B to distinguish between the cube and the cylinder. Depending on their orientation, it was possible to rigidly explore them with the knuckles, but not in all locations of the box. For this reason, we plan to investigate a solution to provide pilots with some information related to rough shape changes in objects.

## Conclusion

Several investigations have provided significant insights into the potential of the SoftHand Pro platform for effectively addressing the limitations of prototype versions and other prosthetic solutions. These studies included able-bodied individuals and transradial amputees. Works since [9] have been instrumental in improving the SHP hardware and software, as well as the aesthetics. Our participation in CYBATHLON and changes in the race rules and tasks from 2016 to 2020 accelerated the development of our prosthesis. For instance, smaller objects were included and thus, augmenting the necessity of performing more precise grips. Accordingly, the size and weight of the prosthesis were reduced with a consequent improvement of the autonomy and aesthetics of the platform. Furthermore, the challenges faced in *Cybathlon 2020 Global Edition* inspired the future the inclusion of additional features and components, such as sensory feedback or active rotational wrist. In summary, the SHP platform showed high functionality with control simplicity. The latter permits the implementation and selection with two of the most common control modalities in the market (i.e. body-powered and direct myoelectric control) according to the user. Lastly, all pilots’ feedback and experience in CYBATHLON have been and continue to be essential for the development of our prototype, especially due to the important contribution that interaction contact forces have in the real use of our technological platform.

## Data Availability

All relevant data are within the paper.

## Appendix A: Task descriptions for Cybathlon 2016

- *#1 Puzzle:* The task was to transfer a 3 x 3 grid of square wooden bases, each with differently shaped “handles” from one puzzle frame to another. The pieces could only be lifted by the handle and the handle could only be manipulated using the prosthetic terminal device. The handles varied in shape, size, texture, and weight to favor different grips postures.
- *#2 Wire loop:* The task was to move a wire loop from one end of a metal wire “course” to another. The wire loop is conductive and any contact with the wire course, with the exception of “safe zones” at the start and finish, resulted in task failure. The course contained 90° turns, diagonal turns, and curves, and the wire loop could be guided only by the prosthetic arm.
- *#3 Shelf and tray:* At the start of the task, many items used to set a breakfast table were arranged on a set of shelves or in drawers. A tray was also provided. Many of these items could only be handled with the prosthetic device. It was required that all of the items, including the tray, be carried over a ramp, through a closed door, down a ramp and set on a table. The Pilot was allowed to do as many trips as needed. Finally, one of the items was a lightbulb in a box, which had to be removed from the box and screwed into a table lamp using only the prosthetic device.
- *#4 Breakfast table:* Several elements of meal preparation were set on a table. This task could be completed using either hand/arm for any part of the task. The components of the task were opening a water bottle, opening a jar, unwrapping a sugar cube, cutting a loaf of bread, and using a can-opener to open to a can.
- *#5 Hang-up:* A clothesline was set up next to uneven terrain. On the clothesline were two clothes hangers and two clothespins. Nearby, was a hamper with a t-shirt, button-up blazer, and zip-up jacket. The Pilot had to pin the shirt to the line, manipulating the pins with his prosthetic arm only, and close and hang both jackets using the hangers (either or both arms could be used for the jackets).
- *#6 Carry:* At the start of this task, objects of various sizes and weights were placed near the bottom of a 3-step staircase. The Pilot had to carry the objects up the stairs, over flat ground, down stairs and place them on a table. The Pilot could make as many trips as desired. Objects included soccer and footballs, watering can, water crate, large box, and large bag ranging in weight from roughly 400 grams to nearly 5 kg.

## Appendix B: Task descriptions for Cybathlon 2020 Global Edition

- *#1 Breakfast:* This task evaluates the ability to use kitchen utensils (e.g. cutlery, can opener etc.), which is critical for self-sustained living. It consist of the preparation of a breakfast table, including cutting bread, unwrapping a piece of chocolate, opening a bottle, a jam jar and a can, and lighting a candle with a match. It involves tasks typically performed by a dexterous bimanual interaction and delicate objects that require a precise control of the grip force.
- *#2 Laundry:* Hanging laundry and clothes manipulation requires a distinct set of fine motor skills and it is a practical task for arm prosthesis daily use. In this task, a hooded sweater must be taken from a coat rack, put on and the zipper must be closed and then, taken off. Clothes that are placed in a clothes hamper need to be hung up on a clothes line by using hangers and blue clothes pegs. Two buttons of a blazer need to be closed and shoes must be tied.
- *#3 Clean sweep:* This task challenges the ability of a technology to cope with a diversity of objects requirements. The objects included and related grip types were chosen based on literature and their relevance in daily life. Besides the ability to use different grip types, the ability to maintain grips during postural changes of the wrist/arm and the control of grip force are tested in this task through the holding and releasing phases. In this task, pilots are asked to grasp and move blue objects individually and in a predefined order from their initial position on the table surface to a target position on the neighbouring table.
- *#4 Home improvement:* The ability to use tools is fundamental to complete maintenance work at home. In this task, various tools and objects (mostly blue objects) are used to complete crafting tasks. Among them, a blue light bulb must be grasp from a table, carried to a floor lamp and screwed into a socket until lit.
- *#5 Haptic box:* In this task evaluates the need of sensory feedback in arm prosthesis. Six objects of specific shape, texture and compliance must be identified without visual feedback. Inside each box, a single object is tightly attached to the bottom of the box such that it cannot be lifted or removed. Objects are presented in a random order. The pilots only rely on sensory feedback from the prosthesis (e.g. sounds, vibrations at the socket, haptic feedback from the terminal device) to solve the task.
- *#6 Stacking:* Maintenance of a tight grip during postural changes of the arm (e.g. pronation and supination of the forearm, elbow flexion and extension) is challenging for prosthetic hand users, but is relevant in many situations. Here, the pilots must turn blue caps and stack them to a vertical pyramid.
